# The public health effects of interventions similar to basic income: a scoping review

**DOI:** 10.1016/S2468-2667(20)30005-0

**Published:** 2020-02-27

**Authors:** Marcia Gibson, Wendy Hearty, Peter Craig

**Affiliations:** a MRC/CSO Social and Public Health Sciences Unit, University of Glasgow, Glasgow, UK; b Improvement Service, Livingston, UK

## Abstract

Universal, unconditional basic income is attracting increasing policy and academic interest. Income is a key health determinant, and a basic income could affect health through its effect on other determinants, such as employment. However, there is little evidence of its potential effects on public health, because no studies of interventions which meet the definition of basic income have been done. However, there is evidence from studies of interventions with similarities to basic income. Therefore, we aimed to identify these studies and to consider what can be learned from them about the potential effects of such interventions on health and socioeconomic outcomes. We did a systematic scoping review of basic income-like interventions, searching eight bibliographic and eight specialist databases from inception to July, 2019, with extensive hand searching. We included publications in English of quantitative and qualitative studies done in upper-middle-income or high-income countries, of universal, permanent, or subsistence-level interventions providing unconditional payments to individuals or families. We sought to identify the range of outcomes reported by relevant studies, and report health, education, employment, and social outcomes. We extracted and tabulated relevant data and narratively reported effects by intervention and outcome. We identified 27 studies of nine heterogeneous interventions, some universal and permanent, and many evaluated using randomised controlled trials or robust quasi-experimental methods. Evidence on health effects was mixed, with strong positive effects on some outcomes, such as birthweight and mental health, but no effect on others. Employment effects were inconsistent, although mostly small for men and larger for women with young children. There was evidence of spill-over effects in studies measuring effects on large populations. In conclusion, little evidence exists of large reductions in employment, and some evidence suggests positive effects on some other outcomes, including health outcomes. Evidence for macro-level effects is scarce. Quasi-experimental and dynamic modelling approaches are well placed to investigate such effects.

## Introduction

There is growing interest in many countries in providing all individuals with an unconditional, regular, basic income. The role of income as a fundamental determinant of health through numerous pathways has stimulated keen public health interest in a policy that would provide equal payments to all.^1^ A full basic income is generally defined as universal, permanent, unconditional, and unaffected by other income.^2^ Some definitions also stipulate subsistence-level payments.^3^ Since the effects of a universal, permanent intervention might differ from those observed in a small, short-term trial, evidence on the full range of potential effects is hard to obtain. Behavioural responses to a permanent basic income might differ from responses to a small-scale time-limited scheme. A basic income implemented at scale might have many spill-over and indirect effects. However, relevant evidence exists from several interventions that are similar to basic income, including quasi-experiments of policy-level interventions affecting large populations.

Although support for basic income has increased, it is still a controversial idea, with many complex arguments for and against. Proponents argue that basic income could reduce poverty and promote equality by paying every citizen the same amount.^3^, ^4^ Work disincentives in benefit systems with high withdrawal rates could be removed, and the cumbersome bureaucracy of means testing could be eradicated,^5^ Moreover, it has been suggested that basic income could free up time for caring, education, community work, and creative or business projects, improve health by reducing stress and stress-related health behaviours, and address employment insecurity, in-work poverty, and potential mass unemployment due to automation.^6^ Critics argue that basic income could disincentivise work and promote economic dependency, is unaffordable, and might not reduce poverty for the poorest.^7^ People with greater needs would not be served by a flat-level payment, and it does not account for variable housing costs.^8^

Several narrative reviews of basic income-like studies in high-income countries have been published, but none use systematic methods to search for and extract data,^1^, ^6^, ^9^ and the composition of the evidence base is unknown. Therefore, we aimed to provide an overview of the existing evidence on basic income-like interventions and their effects, and to consider what can be learned from them about the potential effects of a universal, permanent basic income on health and socio-economic outcomes in a high-income country.

## Methods

### Overview

We did a systematic scoping review The protocol for this study has been published previously.^10^

### Search strategy and selection criteria

We searched eight bibliographic and eight specialist databases for articles published in English from database inception until April, 2017 (iteration 1), November, 2017 (iteration 2), and July, 2019 (searches updated). The search strategy used terms related to basic income, negative income tax, and study design. We also did extensive hand searching (appendix p 4). The database results were uploaded to Endnote, where inclusion and exclusion decisions were recorded. Results were screened by MG and a 10% sample was checked by WH. Sources searched and a detailed description of the searches are available in the appendix (p 4).

No interventions meet all five of the aforementioned criteria for a full basic income. We therefore sought evidence from studies of interventions that meet some of the criteria. We describe these as basic income-like to clarify that they do not meet the all of the criteria, and to avoid the conceptual confusion that arises when effects of basic income are extrapolated from interventions with few characteristics of a basic income. The ability to choose whether to engage in paid employment is arguably the key feature of basic income, and many of the putative effects would not occur if conditions were attached to receipt. Therefore, we included only interventions that provided regular, unconditional payments to individuals or households. To gain insights into the potential effects of other features of a full basic income, we included interventions that also met one or more of the other criteria: universality, permanence, and fixed or subsistence-level payments. We included randomised controlled trials (RCTs), cluster RCTs, quasi-experimental, controlled before-and-after, and qualitative studies in upper-middle-income or high-income countries, aimed at the general population or at low-income groups. To map the available evidence, we recorded studies that reported effects on any outcomes, but only studies reporting labour market participation, health, education, and social outcomes (crime and family functioning) are reported here and included in the synthesis. We defined interventions that gave transfers to a large proportion of the population as quasi-universal. Full details of the inclusion and exclusion criteria are provided in the appendix (p 3).

### Data analysis

A data extraction form was developed and independently piloted on three publications by two reviewers (MG and WH). We extracted intervention and study characteristics and impact data for our focal outcomes, as well as any evidence of spill-over or indirect effects. Duplicate extraction was done on a 25% sample of publications. The manner of reporting data varied across the studies, and we report effects in their original format. Owing to missing data, it was not possible to calculate effect sizes for the included outcomes. In reporting magnitudes of effects, we used the quantifiers supplied by the authors, or in some cases compared the effects with those of interventions aimed at similar outcomes. All effects reported in the text are significant at the 10% level or higher unless otherwise stated. We tabulated intervention and study characteristics, and narratively reported the effects by intervention and outcome.

We did not critically appraise the quality of included studies. We do however comment on major methodological issues with the studies.

### Role of the funding source

The funders of the study had no role in study design, data collection, data analysis, data interpretation, or writing of the report. The corresponding author had full access to all the data in the study and had final responsibility for the decision to submit for publication.

## Results

Excluding duplicates, we found 2890 publications, from which we identified 27 studies of nine interventions, including 11 studies of five historical negative income tax interventions and 16 studies of four contemporary unconditional cash transfer interventions (figure; appendix p 2). One intervention occurred in Iran, with the remainder in North America. Further information on study context, design, and implementation, including which basic income criteria each of the interventions met, is provided in the appendix (p 11). We report the historical and contemporary studies separately, because the differing temporal contexts should be considered when interpreting the results.

**Figure:**
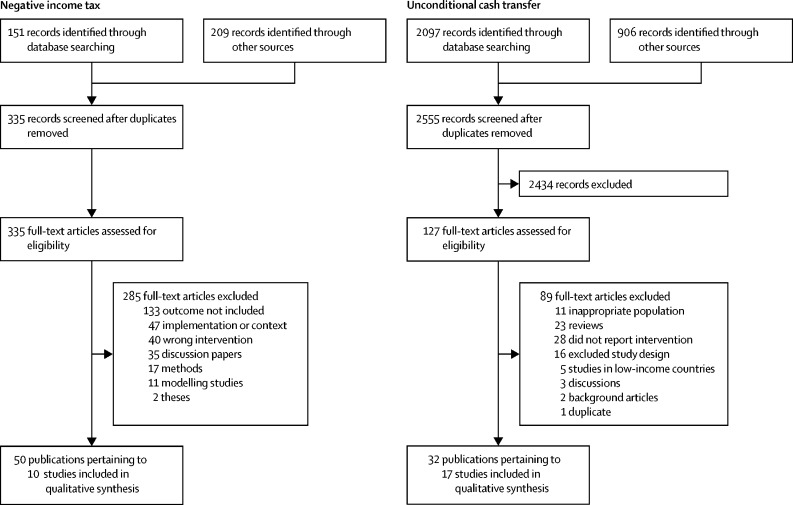
Study profile

### Historical studies

Landmark studies of negative income tax were done in North America in the 1970s with the aim of measuring the work disincentive effects of providing a guaranteed income.^11^, ^12^ For 3–5 years, they provided scattered samples of low-income families with an unconditional, subsistence-level income that was withdrawn at varying rates above varying income thresholds. These studies were done to test differing combinations of these rates and thresholds in New Jersey and Pennsylvania (New Jersey Graduated Work Incentive Experiment [New Jersey]), Iowa and North Carolina (Rural Income Maintenance Experiment [RIME]), Gary in Indiana (Gary Income Maintenance Experiment [Gary]), Seattle and Denver (Seattle-Denver Income Maintenance Experiment [SIME-DIME]), and Winnipeg and rural Manitoba, Canada (Manitoba Basic Annual Income Experiment [Mincome]). The populations, settings, and economic conditions varied widely. Negative income tax was not universal or permanent, and the value fluctuated. Mincome included the rural town of Dauphin as a so-called saturation site, where anyone whose income was below the threshold during the study was eligible, even if they were not in the sample at the start of the study. The methods used in the studies have been criticised; however, some of the criticisms are perhaps overstated (appendix p 9).

With regard to health outcomes, there were small non-significant increases in psychological distress in some SIME-DIME subgroups (table 1).^33^ In RIME there was a mild positive effect overall, associated with plan generosity.^27^ New Jersey reported no effects on psychological outcomes.^24^ Hospital admissions were 8·5% lower across the community in Dauphin, driven by reduced admissions for accidents and mental health conditions.^19^, ^21^ Qualitative data suggested that negative income tax increased respondents' autonomy and self-respect.^17^

**Table 1:** Effects of negative income tax on other outcomes by study

	**Effect**
**Gary Income Maintenance Experiment**	
Adult labour supply: annual hours worked, percentage difference in annual hours worked	Table 2
Teen labour market participation^13^	Low-income boys and girls in generous plans significantly less likely to work*
Marital dissolution	No evidence located
Reading test scores^14^	22-point improvement for younger children,† related to low baseline incomes and length of exposure. No effect on other groups
Remaining in education^15^	Low-income boys and girls in generous plans significantly more likely to stay in school
Academic grade point average^14^	No effect
Days absent^14^	No effect
Birth weight^13^	Increased by 0·3–1·2 lb (136–544 g)† among the highest risk groups
**Manitoba Basic Annual Income Experiment**	
Adult labour supply: annual hours worked, percentage difference in annual hours worked	Table 2
Marital dissolution^16^	Small reduction
Qualitative; explores attitudes towards Mincome, reasons for claiming, and the role of perceived stigma in both of these in saturated site (Dauphin)^17^	Mincome was not stigmatised in the same as way as normal welfare benefits; many claimed Mincome who would not have claimed welfare; Mincome allowed people to respond to changing circumstances while remaining in work; autonomy and dignity were highly valued
Labour market participation in saturated site (Dauphin)^18^	Overall, the reduction in Dauphin was 30% larger than in the scattered site (14·7% *vs* 11·3%),‡ driven by reductions for young people (18·6 percentage points) and single people (16·2 percentage points)
Considering reducing work now or in the future in saturated site (Dauphin)^18^	Qualitative data; 55 of 322 respondents indicated might reduce work due to inability to find work, health problems, education or caring
Starting wage rate on job (for all job vacancies reported in previous week)^15^	Wages 0·66 cents per hour higher in Dauphin (SE 0·39, 90% CI 0·01 to 1·31)§
Wage rate on job (for people hired in the past 4 months)^15^	Wages 0·17 cents per hour higher in Dauphin (SE 0·16, 90% CI −0·10 to 0·44)
Percentage of businesses that received job applications in last four months^15^	12·2% fewer applications in Dauphin (SE 0·1747, 90% CI −0·4104 to 0·1672)
Percentage of businesses with no new employees in last four months^15^	18·6% more Dauphin businesses recruited no new employees (SE 0·0692, 90% CI 0·0718 to 0·2995)‡
Hours per week for new employees^15^	0·07 fewer hours per week in Dauphin (SE 2·69, 90% CI −4·52 to 4·37)
Hours per week in vacant positions^15^	2·76 fewer hours per week in Dauphin (SE 1·62, 90% CI −5·43 to −0·10)§
Total hospital separations (hospitalisation per 1000 people) in saturated site (Dauphin)^19^	8·5% lower than control group,‡ mostly driven by reductions in admissions for accidents or injuries and mental health diagnoses
Overall physician claims and physician claims for mental health diagnoses in saturated site (Dauphin)^19^	Similar pattern to hospital findings is reported†
Percentage progressing to Grade 11 or 12 high school in saturated site (Dauphin)^20^	Increased from 81% to 99% in Dauphin site, decreased from 99% to 90% in control site;* qualitative evidence of peer effects on decision to remain in school
Low birthweight in saturated site (Dauphin)^21^	No effect
Marital dissolution in saturated site (Dauphin)^21^	No effect
**New Jersey Graduated Work Incentive Experiment**	
Adult labour supply: annual hours worked, percentage difference in annual hours worked	Table 2
Teen labour market participation^22^	Large reductions for experimental teens
Marital dissolution^23^	No effect
Level of education completed, school enrolment, and college attendance^22^	25–30% increase in school completion for teens on medium-generosity plans, 6–12 months more education at end of study*
Anomy scale, Control of future scale, Community Efficacy Scale, Psychosomatic and Nervous Symptoms Scale, Self-Esteem Scale, Worry Items, Quality of Life, General happiness, and Feeling of nothing to do^24^	No effect
Household head's and spouse's number of chronic conditions, number of work days lost, number of days spent in a hospital, and number of physician visits^25^	No effect
Children's per capita number of chronic conditions, per capita number of days spent in bed, per capita number of visits to a physician, and whether any child has spent at least one night in a hospital in the year previous to the interview^25^	No effect
**Rural Income Maintenance Experiment**	
Adult labour supply: annual hours worked, % difference in annual hours worked	Table 2
Teen labour market participation^26^	Reductions for all groups, but only significant for white individuals from North Carolina (66% fewer hours per quarter)*
Marital dissolution^27^	No statistically significant effect
Mean Adequacy Ratio of 10 vital nutrients^28^	3·56% higher for North Carolina experimental group;‡ no difference for relatively affluent Iowa sample
Self-report delinquency scale; how many times in last 2 years committed theft, received stolen property, trespassed, committed assault, extortion, or used marijuana or other narcotics; another scale developed to take account of seriousness of offences^27^	Crime higher in low guarantee groups, but substantially lower in high guarantee groups. Only significant for one group.
School attendance^27^	Younger children in North Carolina site; 30·5% reduction in absenteeism;† no effect on other groups
Academic grades and Standardized Achievement Test score^27^	Younger children in North Carolina site; 18·9% improvement in SAT scores, a 6·2% increase in Grade Point Average;† no effect on other groups
Health service use: annual contacts with hospitals, clinics, private physicians, and dentists; whether a family member visited a specialist; cash expenses by the family for doctor, hospital, drug, and dental bills; and whether a family possesses medical insurance^27^	Small and inconsistent effects
Health: work lost due to illness, the presence of a chronic health impairment, and whether this condition limits the amount or type of work practiced by the individual^27^	Small and inconsistent effects
Psychological well-being; scales similar to New Jersey^27^	Mild positive effect for adults and teens across sample. Significance varies across subgroups
**Seattle-Denver Income Maintenance Experiment**	
Adult labour supply: annual hours worked, % difference in annual hours worked	Table 2
Teen labour market participation^29^	Male teens worked 4·63 h fewer per week,‡ and female teens worked 2·78 h fewer§
Marital dissolution^30^	Initial analysis suggested large negative effect; later reanalysis found no effect
Achievement scores, academic grades, and absence rates^29^	No effects
Remaining in school^31^	11% more likely to complete high school*
Number of work days lost due to illnesses; number of hospital stays; number of days hospitalised in the last 2 years; number of work days missed in the last 6 months; presence of a functional limitation on doing household tasks; presence of a chronic condition that limits activities of daily living or market work; the duration of the chronic condition; a mental health index; self-perception of overall health^32^	No effects
Psychological distress: “a close variant of the Macmillan Health opinion survey index”^33^	Very small increases for some subgroups
Income of SIME participants 40 years after intervention^34^	$1800 per annum less than controls†
Labour force participation of SIME participants 40 years after intervention^34^	3·3% less likely to be in work†
Disability benefit claims and percentage of successful claims of SIME participants 40 years after intervention^34^	6·3% more likely to claim‡, but less likely to be successful
Mortality of SIME participants 40 years after intervention^34^	No effect
Impacts on above outcomes for children of SIME participants 40 years after intervention^34^	No effects
Impact on labour market behaviour for sample enrolled for 20 years^35^	Reductions no greater for husbands or second earners. Larger effects for single parents
Labour supply differences between 3-year and 5-year samples; reanalysis of data accounting for variations in co-intervention duration^36^	Contrary to previous analyses, finds no significant difference in labour supply between men in 3-year and 5-year samples

* Significance not reported.

† Significant at the 5% level.

‡ Significant at the 1% level.

§ Significant at the 10% level.

A large increase in birthweight (0·3–1·2lb) for high-risk groups in Gary was attributed to improved maternal nutrition.^37^ There was no effect on birthweight in Dauphin, where the whole community was included in the analysis.^21^ New Jersey and SIME-DIME found no impact on measures of health service use.^25^, ^32^ There was a large reduction in hospital admissions in Dauphin, potentially due to spill-over effects.^19^, ^20^ Nutrition improved in RIME's impoverished North Carolina site, but there was no effect in affluent Iowa.^28^ No effects were found on a range of chronic conditions and health-related limitations in New Jersey^25^ or SIME-DIME.^32^ There was no effect on mortality in SIME-DIME 40 years after the intervention.^34^

Several analyses of data on labour market participation (LMP) from studies of negative income tax have used different statistical methods and approaches to defining the sample. We present the range of effects reported by Hum and Simpson (table 2).^41^ Across the studies, husbands' annual hours worked were 1–9% lower in groups that received payments than in those that did not. Effects for wives (3–33%) and single parents (7–30%) were larger than those for men. Few of these effects were significant. Men reportedly spent longer looking for work,^40^, ^42^ and women spent more time on domestic tasks.^12^ Intervention duration had no effect,^35^ but an analysis of administrative data for SIME-DIME respondents 40 years after the intervention found that LMP was reduced by 3·3%.^34^

**Table 2:** Effects of negative income tax on annual hours worked by study

	**Male heads**	**Second earners**	**Female heads**
**New Jersey Graduated Work Incentive Experiment**			
Keely (1981)^38^	−116 (−7%)	−75 (−33%)	..
Robins (1985)^39^	−34 (−2%)	−56 (−25%)	..
Burtless (1986)^40^	−21 (−1%)	−56 (−25%)	..
**Rural Income Maintenance Experiment**			
Keely (1981)^38^	NR (−9%)	NR (−29%)*	..
Robins (1985)^39^	−56 (−3%)	−178 (−28%)	..
Burtless (1986)^40^	−56 (−3%)	−178 (−28%)	..
**Seattle-Denver Income Maintenance Experiment**			
Keely (1981)^38^	−147 (−8%)*	−139 (−21%)*	−155 (−15%)*
Robins (1985)^39^	−113 (−7%)*	−141 (−21%)*	−163 (−16%)*
Burtless (1986)^40^	−144 (−8%)	−107 (−17%)	−85 (−9%)
**Gary Income Maintenance Experiment**			
Keely (1981)^38^	−80 (−5%)	−9 (−3%)	−102 (−28%)
Robins (1985)^39^	−35 (−2%)	−58 (−20%)	−37 (−10%)
Burtless (1986)^40^	−114 (7%)	14 (5%)	−112 (−30%)
**All US experiments**			
Robins (1985)^39^	−89 (−5%)	−117 (−21%)	−123 (−13%)
Burtless (1986)^40^	−119 (−7%)	−93 (−17%)	−133 (−17%)
**Manitoba Basic Annual Income Experiment**			
Hum and Simpson (1993)^41^	−17 (−1%)†	−15 (−3%)	−79 (−7%)

NR=not reported.

* Statistical significance at the 5% level or lower. In some cases, statistical significance is not reported or is mixed (the result is an average of several results, some of which are significant). Burtless (1986)^40^ does not report statistical significance.

† Includes single individuals (21% of all men in sample).

A difference-in-difference analysis of a small, potentially biased sample from Mincome's saturated site in Dauphin found a larger reduction in LMP in the saturated site than the dispersed site. Qualitative analysis found so-called community context effects, including increased acceptability of receiving payments.^18^ Qualitative evidence suggested that negative income tax allowed people to respond flexibly to changing circumstances (eg, health problems, caring, and education), because they could remain in work without losing benefits, and that the dignity and autonomy thus afforded were highly valued.^17^ A further difference-in-difference analysis of Mincome data from a survey of business owners found that employers received fewer applications for new posts in Dauphin than the control sites, and wages for new vacancies were higher (CAN$0·66 per hour).^15^

RIME and Gary reported substantial improvements in several educational outcomes for younger and more disadvantaged children.^14^, ^43^ SIME-DIME found no effects on measures of educational performance.^29^ Teens whose families received payments in SIME and Dauphin were more likely to complete high school and less likely to work than those who did not receive payments,^21^, ^29^, ^31^ as were some groups in New Jersey,^22^ Gary,^13^ and RIME.^26^ Qualitative evidence from Dauphin suggests financial considerations and peer effects influenced decisions to remain in education.^20^

There were no effects on marital dissolution in SIME-DIME^30^ or New Jersey.^23^ Small reductions were found in RIME^27^ and Mincome^16^ but not in Dauphin.^21^ Measures of teen offending showed mostly non-significant reductions for some groups in SIME-DIME^44^ and RIME,^27^ but the SIME sample was very small. Both results in RIME were related to plan generosity.

### Contemporary studies

The Alaska Permanent Fund has paid dividends as an annual lump sum to all Alaskan residents from the state's oil revenues since 1982. The payments are not affected by other income, but they fluctuate and are less than subsistence level. However, they are substantial at household level, with individual payments ranging from US$1000 in 1996 to $2072 in 2015.^45^

The Iranian Targeted Subsidy Plan has paid all individuals a fixed monthly sum since 2010 to compensate for the abolition of fuel subsidies. Initially this payment was above subsistence-level, but it was very quickly eroded by inflation.

Some Native American nations have been running casinos since the mid-1990s and distributing dividends from the revenues to all tribal members. The payments are permanent and universal within the tribe. The value varies across tribes, from below subsistence-level to well above, but is often substantial. Childhood payments accrue and are paid as a lump sum on adulthood. The Great Smokey Mountains Study (GSMS)^46^ is one of a number included in this Review.

The Ontario Basic Income Pilot (OBIP) was a negative income tax (unconditional, subsistence-level, and withdrawable). It began in 2018 but was terminated early upon a change of provincial government. Some qualitative data have been collected from participants.

Between 2000 and 2006, mortality increased by 13% in the weeks following Alaska Permanent Fund receipt; 8% was attributed to increased substance abuse and the remainder to displaced activity, with a concomitant reduction 4 weeks later.^47^ Eastern Cherokee accidental mortality risk doubled following dividend receipt, with 50% of deaths involving motor vehicles. Qualitative evidence indicated payments were associated with substance abuse and vehicle purchase, particularly following receipt of the first large cheque at 18 years old.^48^ Alaska Permanent Fund and tribal dividends are large lump sums paid once or twice per year.

Low birthweight in Alaska was 0·7 percentage points lower, and birthweight increased by 17·7 g for every US$1000 increase in income, apparently owing to longer gestation.^49^ Among children born to middle-income Alaskan families ($25–75k per annum) in 2009–11, the proportion of obese 3 year olds decreased by 22·4%, but no effect was observed for other income groups. In a simulation model, every dollar paid in dividends led to 20–92 cents lower health-care expenditure.^50^ In GSMS, dividends were associated with reduced body-mass index and probability of obesity, but both measures were higher for adolescents who were poor at baseline.^51^

Several GSMS analyses reported positive effects on a range of child and adolescent personality traits and mental health outcomes in successive waves of data following dividend implementation.^52^, ^53^ Improved adult mental health was posited as a mechanism underlying better child outcomes, as were improved parental supervision,^46^, ^54^ improved parental and parent-child relationships,^52^, ^54^ and fewer delinquent peers in adolescence.^63^ There was evidence for a cumulative effect of exposure,^53^, ^54^ and effects were often stronger in the most high-risk groups.^46^, ^52^

Improved adult mental health was potentially due to decreased financial stress.^54^ A qualitative tribal dividend study^55^ also reported that reduced financial stress led to improved mental health. Qualitative evidence from two studies of OBIP^56^, ^57^ indicated that escaping intrusive bureaucracy led to reduced stress, and greater food security was linked to amelioration of other health conditions, including depression, fibromyalgia, and coeliac disease. Payments were used for medications, dental treatment, and optical needs. Some of these effects were due to the higher value of OBIP payments (than normal benefits).

One tribal qualitative study reported that affordability of physical activity and healthy food increased after per capita dividend payments were introduced. However, tribal elders reported that unhealthy eating, substance abuse, and gambling increased.^55^ Qualitative evidence of increased substance abuse was reported by another tribal dividend study,^58^ possibly driven by receipt of large lump sums. A difference-in-difference analysis of Alaskan data found that substance abuse-related crime increased in the 4 weeks following receipt of annual payments.^59^ In GSMS, there was no effect on several child health outcomes (accidents, allergies, headaches, and eczema).^51^

Three studies reported LMP effects of the Alaska Permanent Fund (table 3). One synthetic control study found no long-term effect on LMP, but an increase in part-time working and a small decrease in hours worked, driven by more women working part time (22% increase).^45^ A difference-in-difference study using data from 2005–15 found that annual hours worked decreased by 182 h for men, 106 h for single women, and 151 h for married women. These changes correspond to an 11% decrease for men and 12% for married women.^62^ A further difference-in-difference study analysed the short-term effects of payment receipt on LMP, finding that male LMP increased by 1·6%, but women's average weekly hours decreased, particularly if they had young children (−1·96 h weekly). The net aggregate effect was a 0·2% annual reduction in labour supply.^61^

**Table 3:** Effects of unconditional cash transfer on all outcomes by intervention

		**Effect**
**Alaska Permanent Fund**		
Low birthweight^49^		0·7 percentage points lower;* birthweight 17·7 g higher for every $1000 increase in income*
AGPAR score: mean, proportion with low score^49^		0·063 higher;* low AGPAR score 0·4% lower*
Prenatal care^49^		Prenatal care began 2·23 days earlier;* no effect on number of visits
Mortality^47^		13% increase among urban Alaskans immediately following annual payment receipt;† 8% of this attributable to increased substance use,† the remainder to an activity displacement effect
Probability of child obesity at 3 years of age^50^		4·5 percentage points lower per $1000 additional dividend;* corresponds to a 22·4% reduction in number of obese 3-year-old Alaskans
	Annual household income <$25 000	No effect
	Annual household income $25 000–75 000	4·5 percentage point reduction in probability of obesity; 22·4% fewer cases; significant but significance not reported
	Annual household income >$75 000	No effect
Employment rate^45^		No effect
Labour force participation^45^		No effect
Part-time employment rate^45^		17% increase*
	Men	No effect
	Women	22% increase‡
Hours worked last week^45^		0·617 decrease
Income inequality (Gini coefficient, relative mean deviation, and Thiel's Entropy Index)^60^		Gini Coefficient 0·21* higher, relative mean deviation 0·13 higher,‡ and Thiel's Entropy Index 0·36 higher* in the long term
Number of hours worked in reference week^61^		0·59 h (SE 0·253) decrease per $1000 increase in dividend payment‡
	Men	0·244 h (SE 0·346) decrease per $1000 increase in dividend payment
	Women	0·913 h (SE 0·335) decrease per $1000 increase in dividend payment;* 1·96 h (SE 0·848) decrease for women with children younger than 5 years‡
Whether respondent employed in reference week^61^		0·6% (SE 0·006) increase per $1000 increase in dividend payment
	Men	1·6% (SE 0·007) increase per $1000 increase in dividend payment‡
	Women	0·4% (SE 0·009) decrease per $1000 increase in dividend payment
Crime 1 day after Permanent Fund Dividend receipt^59^		
	Noise violations	No effect
	Property crime	No effect
	Substance abuse-related crime	6·16 more incidents (SE 1·964)*
	Violent crime	No effect
	Medical assistance to other agencies	No effect
Crime 4 weeks after Permanent Fund Dividend receipt^59^		
	Noise violations	No effect
	Property crime	Average 8% fewer incidents per day; significant but level not reported
	Substance abuse-related crime	Average 10% more incidents per day; significant but level not reported
	Violent crime	No effect
	Medical assistance to other agencies	Average 9% more incidents per day; significant but level not reported
Annual hours worked (triple difference comparison with all states)^62^		
	Men	−182 h per year (SE 3·182)*
	Single women	−106 h per year (SE 3·561)‡
	Married women	−151 h per year (SE 3·835)‡
**Iranian targeted subsidy plan**		
Probability of low-income labour market participation^63^		
	Men	No effect
	Women	Increased by 7%*
Low-income hours worked (fixed effects, timing of participation)^63^		
	Men	Increased by 0·069 h per week*
	Women	No effect
Hours worked^63^		
	Waged	Increased by 0·066 h per week‡
	Self-employed	Increased by 0·082 h per week‡
	Waged and self-employed	Increased by 0·050 h per week, but not significant
**Ontario Basic Income Pilot**		
Recipients' accounts of how Ontario Basic Income Pilot affected them^56^		Improvements reported in many areas, including ability to explore different options and cope with various personal circumstances, long-term planning, improved diet leading to better health, paying off debt, dignity, and social interaction
Recipients' accounts of how Ontario Basic Income Pilot affected them^57^		Improvements reported in many areas including ability to plan, ability to take up work that fits around personal circumstances (particularly health issues), and work incentives; not having to deal with intrusive bureaucracy and removal of risk of sanctions was reported to reduce stress
**Tribal dividends**		
Accidental mortality^48^		Increase in dividend payment months; risk ratio 2·62, 95% CI 1·54–4·47
	Substance abuse (qualitative)	Ethnographic evidence suggested young people often spent lump sum dividends on motor vehicles and substances
Unemployment^64^		
	Native Americans only	−3·13%‡
	All	−2·09%‡
Labour force participation^64^		
	Native Americans only	−7·22%*
	All	−3·22%‡
Per capita income		
	Native Americans only	$3944·79*
	All	$3141·17*
Qualitative; community perceptions of effects of casinos^58^		No effect on adult labour force participation; some reports of young adults living off their dividends; reports of increased substance abuse, but relevant personnel reported drops in driving under the influence, robbery, petty crimes, and increased participation in adult education; some conflict over eligibility for dividends (ie, tribal membership)
Young adult obesity^51^		2–4% decrease in probability of obesity at 21 per $5000 per annum higher initial income,* but increased for those in poverty before dividends
Young adult body-mass index^51^		0·6 lower at 21 per $5000 per annum higher initial income,‡ but higher for those in poverty prior to dividends
Child health: accidents, allergies, headaches, and eczema^51^		No effect
Psychiatric disorders among children and adolescents—emotional (anxiety or depression), behavioural (conduct or oppositional defiant disorder), and substance abuse disorder^53^		Odds of any disorder lower for Native American young adults (OR 0·66, 95% CI 0·48–0·90); reductions limited to alcohol and cannabis abuse
Child and Adolescent Psychiatric Assessment Symptom Score^46^		For those who exited poverty, score fell to that of never-poor children (4·28 to 2·90)
Emotional and behavioural distress^52^		−37% (SE 0·104) of a SD and −23% (SE 0·104) of a SD; significant but level not reported
Trait conscientiousness^52^		+25% (SE 0·128) of a SD; significant but level not reported
Trait agreeableness^52^		+37% (SE 0·147) of a SD; significant but level not reported
Trait neuroticism^52^		+0·38% (SE 0·141) of a SD; significant but level not reported
Parental mental health (whether one or both parents sought mental health support)^54^		Cumulative reductions in probability 2, 3, and 4 years after dividends began‡
Maternal and paternal labour force participation rate^54^		No effect
Educational attainment (years of completed education at age 21)^54^		1·1 years longer in education for children in poverty at baseline;* no effect on those not in poverty
Finished high school by 19 years of age^54^		+39% probability for children in poverty at baseline;* no effect on those not in poverty
High school diploma or general equivalency degree by 19 years of age^54^		No effect
School attendance (days in previous quarter)^54^		3·85 additional days per quarter for children in poverty at baseline;‡ no effect on those not in poverty
Criminal arrest figures^50^		
	Young adult	22% less likely to have been arrested at 16–17 years of age;‡ 7% less likely to have dealt drugs at 21 years of age†
	Adult	3·9% reduction in probability of maternal arrest;‡ 11% reduction in probability of paternal arrest*
Marital status^52^		No effect
Parent-child relationship quality^52^, ^54^		Maternal relationships improved by 4%;* no significant improvement for fathers
Parental supervision^46^, ^52^, ^54^		3–5% improvement* for mothers and fathers
Qualitative; mechanisms linking casinos to health^55^		Changes in tribal economy, built environment, and social landscape were identified as mechanisms connecting casinos and health; reduced financial stress and improved health behaviours were linked to higher incomes; some reports of payments financing substance abuse and dependency among young people

SE=standard error.

* Significant at the 1% level.

† Significant at the 10% level.

‡ Significant at the 5% level.

No effect on maternal or paternal LMP was observed in the GSMS tribal dividend study.^54^ A study of all Native American nations using data from 1990–2000 found a decrease in the unemployment rate, but economic inactivity increased.^64^ Two qualitative tribal dividend studies reported decreased economic activity, particularly in some young people,^55^, ^58^ but administrative data in one showed no change in LMP.^55^ In the first year after the Iranian subsidy reform, low-income male LMP and hours worked did not change, but the probability of LMP for low-income women and hours worked for self-employed men increased.^63^

Qualitative data from two studies of OBIP echoed the Dauphin findings on flexibility and choice. The income floor provided by the responsive negative income tax-style payments allowed people to cope with fluctuating health conditions and precarious employment, to explore other career options, retrain, volunteer, or reduce very long working hours.^56^, ^57^

A tribal dividend study^54^ found that educational participation increased by up to a year for individuals who received payments and who were most disadvantaged before their introduction compared with those who did not receive payments. Qualitative evidence indicated that tribal dividends were associated with increased participation in adult education.^58^

Several GSMS papers reported positive effects (from 25% to 50% of a standard deviation) on parental supervision,^46^, ^52^, ^54^ parent-child relationships,^52^, ^65^ and parental relationships and no effect on marital status.^52^ Alaskan mothers of young children spent more time at home, and OBIP respondents reported increased time with children and social interaction.^56^, ^57^

Adolescent and parental offending decreased among the Eastern Cherokee, with reduced adolescent arrests and drug dealing.^54^ Social conflict due to disputed eligibility was reported in a qualitative tribal dividend study.^58^ Income inequality increased in Alaska,^60^ due to the regressive nature of the Alaska Permanent Fund.^66^ 10% more substance-abuse related crime was reported per day in the month following Alaska Permanent Fund receipt, but 8% less property crime was reported. The effect of these short-term increases on annual crime rates was minimal. The authors estimate the net financial impact ranges from a $329 000 reduction in expenditure to a $3·4 million increase; the upper bound of this estimate represents an annual cost of $16 per capita.^59^

### Spill-over effects

There was evidence of spill-over or indirect effects in universal and quasi-universal interventions. The Alaska Permanent Fund increased demand and consumption, leading to a short-term increase in Alaskan male LMP,^61^ and possibly countering longer-term LMP reductions.^45^ Average employment and income improved in Native American nations, possibly because non-Native American residents benefited from economic growth.^64^ There was a larger LMP reduction in Dauphin than in the scattered sample, apparently because universal eligibility led to reduced stigma.^18^ There is also evidence that wages increased in Dauphin, possibly due to reduced labour supply.^15^ Increased hours worked by the self-employed in Iran implies greater business activity.^63^ There was no evidence that permanent interventions had stronger effects on LMP.^45^, ^54^

Alaskan mothers' increased time at home seemed to be associated with reduced infant obesity, which is predicted to reduce health-care expenditure.^50^ Changes in Alaskan crime rates also had cost implications.^59^ A large reduction in hospital admissions occurred in Dauphin, although only 30% of residents received supplements,^19^ and there was qualitative evidence of peer effects on young people remaining in education.^20^ The positive effects of tribal dividends increased with duration of exposure, suggesting that a permanent intervention could have cumulative effects.^51^, ^53^, ^54^

## Discussion

To our knowledge, this is the first scoping review that considers the effects of basic income-like interventions in high-income countries on a wide range of health, economic, and social outcomes. We found some robust studies of interventions that were implemented universally or quasi-universally, in some cases permanently. Effects on LMP were inconsistent. In the early studies of negative income tax, where payments covered subsistence but were withdrawable, reductions of up to 9% in male hours worked were observed, but they were attenuated when underreporting was accounted for in Gary and SIME-DIME.^40^, ^67^ Studies of the Alaska Permanent Fund, which does not cover subsistence, reported contradictory findings for men, with two finding null or small positive effects and one finding an 11% reduction in annual hours worked, possibly owing to differences in study design. Larger reductions in LMP for women appeared to be concentrated in mothers of young children in both the historical and the contemporary studies. However, paid maternity leave was not commonly available to women in these studies, and there is evidence of improved maternal and child outcomes when paid leave is provided.^68^ There was some evidence of increased LMP, including soon after receipt of the Alaskan dividend and among women and the self-employed in Iran. Where LMP decreased, it was often replaced by other productive activities, such as education or caring for dependants. We could not discern a relationship between duration, value, or withdrawal rates and the magnitude of LMP effects. Adolescent LMP was lower, and there were some large increases in educational participation. There were also some strong positive effects on educational performance and attendance, although these were less consistent.

In some studies, there were modest to strong positive effects on a number of health outcomes, including low birthweight, infant obesity, adult and child mental health, service use, and nutrition. Some studies suggested mechanisms underlying these improvements, including reduced stress, improved parenting quality, and reduced financial strain. Several studies reported reductions in offending, but the Alaska Permanent Fund was linked to increased substance abuse-related crime and reduced property crime. There did not seem to be any effect on marital dissolution, but one study reported strong positive effects on family relationships, and other evidence suggested parents spent more time with children. Many studies reported stronger effects on health and educational outcomes in more disadvantaged groups. Some of the effects on these outcomes exceed those typically achieved by interventions targeted at such outcomes, such as provision of micronutrients for low birthweight^69^ or higher education expenditure to increase retention.^70^

There is some evidence of spill-over and indirect effects, and of effects on some outcomes strengthening over time. Consumption increases in Alaska stimulated increased demand for labour. One study suggested that employers might be induced to increase wages, and another that economic stimulus might benefit non-recipients. Mental health improvements in Dauphin seemed to reduce service use and benefit the whole community, and the reduced infant obesity resulting from lower maternal employment was projected to realise substantial savings in health-care expenditure. Improved child mental health and educational outcomes in the GSMS appeared to be mediated by reduced parental problems and better parenting, which became stronger over time. Positive effects on outcomes, such as low birthweight and educational participation, could have long-term individual and societal implications, including increased incomes,^71^ improved adult health,^72^ improved late-life cognitive ability,^73^ reduced mortality,^72^ and increased productivity.^70^

Some adverse effects were reported, including increases in accidental mortality and some types of crime related to receipt of large lump sums. Substance abuse was implicated in these increases, a pattern also seen after salary and social security payments.^47^ There were also some qualitative reports of higher substance abuse, perhaps linked to large lump sums for young adults in tribal dividend studies. A review of the effect of cash transfers on consumption of temptation goods in low-income and middle-income countries reported that quantitative studies found unchanged or reduced consumption, but qualitative studies often reported increases, possibly due to salience bias in small communities and multiple respondents reporting the behaviour of a small number of people.^74^

A common argument against basic income, that it will lead to large reductions in employment, is not supported by the evidence reported here. Given the relatively small number of studies, new evidence could emerge that contradicts these findings. However, the findings of this Scoping Review are congruent with reviews of cash transfers in low-income and middle-income countries, which find little effect on adult LMP,^75^, ^76^ positive effects on child labour,^77^, ^78^ health, and a wide range of structural determinants,^79^ as well as economic spill overs with multiplier effects in local economies.^78^, ^80^ Despite contextual differences, it seems plausible that the mechanisms underlying these effects are similar.^20^

Some of the studies were limited by small samples, multiple subgroups, or poor reporting of methods. However, the majority of the quantitative studies are quasi-experiments, and many are large and well designed with population-based samples. All of the included policy-level interventions are funded through sources of so-called windfall revenue (oil or casino dividends or subsidy abolition). A basic income implemented at national level would likely be funded by general taxation, which might have to increase. The implications of funding a basic income through general taxation for its effects are unpredictable. However, the findings are indicative of behavioural responses to unconditional, regular payments. The contexts, interventions, and studies included are highly heterogeneous, but evidence on pathways linking basic income to other outcomes might be applicable in different contexts. The searches were pragmatic and constrained by available resources, but we are reasonably confident that we identified most relevant interventions through our existing knowledge and extensive hand searching of specialist websites.

Many questions regarding the societal effects of implementing basic income at scale remain unresolved. We found no studies that assessed the economic effects of reduced health service use, remaining in education, or reduced offending. Evidence on macroeconomic outcomes, such as productivity, consumption, labour demand, wages, or inflation is either absent or scarce. However, many other social interventions have spill-over effects when implemented at scale, as evidenced by cluster RCTs and quasi-experiments showing the general equilibrium effects of active labour market programmes.^81^, ^82^

To understand higher-level effects, large samples with a high density of recipients exposed to the same intervention are required, as are appropriate comparison samples.^83^ Community-level randomisation, like the cluster RCT underway in Kenya,^84^ permits measurement of spill-over and community effects but is expensive and logistically challenging. Quasi-experiments, such as many of the included studies, allow robust evaluation of the macro-level effects of interventions that affect whole populations or large groups, often using existing data. Dynamic simulation modelling approaches, such as agent-based modelling, might also help to provide insight into the emergent effects of basic income.^85^
