# Supplementary material for: The public health effects of interventions similar to basic income: a scoping review
Source: Lancet Public Health. 2020 Feb 27;5(3):e165–76. doi: 10.1016/S2468-2667(20)30005-0 (PMC7208547; doi:10.1016/S2468-2667(20)30005-0)
Supplement: Supplementary appendix [file mmc1.pdf]

THE LANCET
Public Health

Supplementary appendix

This appendix formed part of the original submission and has been peer reviewed. We post it as supplied by the authors.

Supplement to: Gibson M, Hearty W, Craig P. The public health effects of interventions similar to basic income: a scoping review. *Lancet Public Health* 2020; **5:** e165–76.

Supplementary Appendix

Contents

Search Methods 2
- Historical Negative Income Tax Studies 2
- Contemporary studies 2

Study inclusion criteria 3
- Population 3
- Intervention 4
- Setting 4
- Comparison 4
- Outcomes 4
- Study design 4

Selection of studies and publications 4

Search strategy 6
- Bibliographic database search - first iteration 6
- Bibliographic database search - second iteration 6
- Bibliographic database search - update 7
- Specialist database searches – second iteration 8
- Specialist database searches – update 8

Characteristics of studies 9
- Historical Negative Income Tax Studies (1968-1980) 9
- Contemporary studies 9
  - Iranian targeted subsidy plan (2010-) 9
  - Alaska Permanent Fund (1982-) 10
  - Tribal dividends (1990s-) 10
  - Ontario Basic Income Pilot (2018-19) 10

Basic income criteria 11

References 12

Intervention and study characteristics tables 14
- Supplementary table 4 Negative Income Tax intervention characteristics 15
- Negative Income Tax study characteristics 18
- Contemporary intervention characteristics 19
- Contemporary study characteristics 22
- All outcomes reported by included studies 26

Search Methods

Due to the nature of the literature available, we conducted searching and screening of the historical Negative Income Tax (NIT) and contemporary studies and publications separately. The NIT studies were known to us and included by definition at the outset, but there was a vast literature to identify and screen for specific publications to extract. By contrast, we did not have prior knowledge of all relevant contemporary studies, thus it was necessary to conduct searches in the usual way.

We developed a search strategy using terms related to basic income, negative income tax, impacts, and study design. Five databases were searched using these terms in April 2017. The information scientist and researchers kept a search diary detailing search terms used and databases searched. On completion of screening the initial database searches, it became apparent that there were many contemporary studies, including dividend payments such as the Native American casino studies, which the original search had not identified. We therefore conducted further pragmatic searches of a limited number of databases using narrow search terms to identify contemporary studies. We also searched a number of specialist international development databases. The searches were updated in July 2019, using terms related to both unconditional cash transfers and basic income. A summary of the sources searched is provided in Supplementary table 1.

Historical Negative Income Tax Studies

Multiple research organisations worked on the NIT evaluations, generating publications running into the thousands. We handsearched several relevant sources, including the websites of Mathematica Inc. and the Institute for Research on Poverty, two of the prominent research organisations involved with the evaluations. Both of these sites provide bibliographies of NIT publications, which were also searched. Karl Widerquist[1] provides a comprehensive bibliography of academic publications relating to the NIT experiments, and this was also handsearched. Finally, many of the references identified by the database searches were books which reported the NIT findings. We screened the contents of the books to identify relevant chapters.

Contemporary studies

In addition to screening the original database searches for relevant publications, we conducted further searches of four bibliographic databases and eight specialist international development databases for contemporary studies. We used the search terms “unconditional cash transfer”, “tribal casino”, and “Alaska Permanent Fund”. We also handsearched the journal Basic Income Studies and the websites of the Basic Income Earth Network and the Citizen’s Basic Income Trust. In addition, we had a number of publications belonging to our own collections.

Database/website	Iteration 1	Iteration 2	Update*
Web of Science Collection	✓	✓	✓
PubMed	-	✓	✓
IBSS	-	✓	✓
Ovid Medline	-	✓	-
WorldCat	✓	-	-
Planex	✓	-	-
Copac	✓	-	-
Scopus	✓	-	
EconPapers	-	✓	✓
3ie	-	✓	-
World Bank	-	✓	-
DFiD	-	✓	-
SSRN	-	✓	✓
Socialprotection.org	-	✓	✓
Institute for Poverty Action	-	✓	-
ILO	-	✓	-

*Supplementary table 1 Bibliographic and international development databases searched. *The July 2019 search update prioritised databases in which included studies were previously identified.*

Study inclusion criteria

We sought evidence from interventions in which individuals or households were unconditionally provided with substantial, regular sums of money on a regular basis. The inclusion and exclusion criteria were refined iteratively, and differ slightly from those laid out in the protocol.[1]

While other criteria are often included in definitions of basic income, we deemed universality, permanence, unconditionality, and fixed or subsistence-level payments to be the most important features in terms of understanding the potential impacts of a full basic income. We did not use universality or permanence as inclusion criteria, as there are very few such interventions, and we did not exclude studies with payments which were withdrawn or were below subsistence level, in order to assess the influence of variations in these criteria. The ability to choose whether to engage in paid employment is arguably the key feature of basic income, and many of the putative effects would not occur if conditions were attached to receipt. Therefore, we included only interventions which provided regular, unconditional payments to individuals or households.

We had originally planned to include studies from any country, but we found more contemporary unconditional cash transfer studies than anticipated, many of which were conducted in low-income countries that experience extreme poverty. Since transferability to the current context is likely to be limited, we narrowed the focus to studies conducted in lower middle, upper middle, and high-income countries (LMIC, UMIC, and HIC respectively). Subsequently, we narrowed this criterion further to exclude studies from LMICs, because it became apparent that transferability was still limited by contextual differences, and because many of these studies are included in existing reviews.

Population

The population of interest was the general population, low income, or unemployed people. This meant that targeted social assistance programmes aimed at people on low incomes were included, as long as there were no other eligibility criteria. In a change to the published protocol, interventions aimed at particularly vulnerable groups, including children, elderly people, people in extreme poverty, widows, orphans, disabled people, and people suffering from particular diseases were excluded, as these groups are not representative of the general population.

Intervention

We included programmes involving regular cash transfers, whether this comprised a fixed sum or fluctuated in response to other income. One-off cash payments were excluded. Cash payments which were conditional upon labour market participation, health service use, educational attendance/attainment, or any other behavioural requirement were excluded.

Setting

Using the World Bank classification of countries by income level, studies conducted in low and lower middle income countries were excluded. Hence, we included studies conducted in upper middle and high income countries.

Comparison

Studies with comparison groups receiving no intervention were included, as were studies which involved different study arms providing variants of the intervention in addition to a group receiving no intervention. There were some studies that compared unconditional cash transfers with conditional payments. Since our interest is in unconditional payments compared with existing systems, these were excluded. 'Fuzzy' unconditional interventions, in which some participants either believed they were subject to conditions, or were in fact subject to conditions, were also excluded.

Outcomes

There were no restrictions on study inclusion by outcome reported, but we did not extract impact data on all reported outcomes. Only effects on specified outcomes are reported and included in the synthesis.

Study design

Evaluation studies with a comparison group, including RCTs and cluster RCTs, quasi- experimental studies (difference-in difference, synthetic control, interrupted time series, regression discontinuity, and instrumental variable), and before and after studies with a separate control or comparison group were included. Process evaluations and qualitative studies were included. Publications that provided information on the role of study or intervention design, implementation or context in relevant completed studies were also included.

General commentary and advocacy publications which did not include empirical analysis were excluded. Simulation studies that use existing data to model the predicted effects of policy changes were also excluded.

Selection of studies and publications

The bibliographic database search results were uploaded to Endnote, and the NIT references were managed as described below. Inclusion decisions were recorded and reasons for exclusion were noted in Endnote. Search results were screened by one reviewer and a 10% sample was checked by a second. Flow charts are provided in the main paper.

We did not manually upload all of the NIT references identified in the sources described above. Instead, the screening criteria described below were applied prior to uploading selected references to an Excel spreadsheet for more detailed screening. References duplicated in any source were also excluded at this stage. We then collated the findings from the electronic database searches, our own collections, and the handsearches described in order to identify suitable papers for extraction.

Many NIT publications reported the same outcomes, or interim findings from early in the studies. In addition, the initial findings for some outcomes were subsequently re-analysed. We adopted a pragmatic approach to selecting publications for detailed extraction. We prioritised papers reporting outcomes of particular interest to this review, but we recorded all outcomes reported by the publications uploaded to Excel. The criteria for selecting references for manual upload are described below.

- We did not upload impact papers which reported early or interim findings
- The initial findings on labour supply and marital dissolution were later called into question, and several papers reporting differing interpretations were published. We uploaded these
- Papers reporting any outcomes other than labour supply or marital dissolution were uploaded
- Papers focusing on methods, design, or contextual issues of particular relevance to our research questions were uploaded.

The five original NIT evaluations generated hundreds of publications and analyses. We defined each intervention, and all publications reporting its impacts, as a single study. Later analyses of NIT study data conducted by researchers not connected with the original studies were defined as discrete studies.

Search strategy

Bibliographic database search - first iteration

"basic income"
"citizens basic income"
"citizens income"
"guaranteed annual income"
"income maintenance"
"negative income tax"
"universal basic income"
"universal income guarantee"
"universal allowance"
"guaranteed income"
"income-maintenance programs"

Implement*
Impact
Trial
Consequence
Intervention*
Result*
Evaluat*
Experiment*

Web of Science 7.3.17

With phrase above and

Experiment or impact

Planex

"basic income"

"citizens income"

Scopus 28.3.17

"basic income"

AND

Experiment or impact

"basic income"

AND

Trial

All terms above in appropriate combinations

Copac 5.4.17

"basic income"

WorldCat 5.4.17

"basic income"

Bibliographic database search - second iteration

6/11/17

PubMed
WoS Core collection
IBSS
Ovid Medline

“unconditional cash transfer”

23/11/17

PubMed
WoS Core collection
IBSS
Ovid Medline

“Tribal casino”

1/12/17

PubMed
WoS Core collection
IBSS
Ovid Medline

“Alaska Permanent Dividend Fund”

Bibliographic database search - update

4/7/19

WoS Core Collection
IBSS
PubMed

"Cash transfer"

4/7/19

WoS Core Collection
IBSS
PubMed

"basic income"

30/7/19

WoS Core Collection
IBSS
PubMed

"tribal casino"

30/7/19

WoS Core Collection
IBSS
PubMed

“Alaska Permanent Fund”

26/7/19

WoS Core Collection
IBSS
PubMed

Iran energy subsidy reform

Specialist database searches – second iteration

**Database**	**Search parameters**	**Date**
**EconPapers**	"unconditional cash transfer" in title or keywords	02/11/2017
**3ie**	free text search "unconditional cash transfer"	02/11/2017
**World Bank**	free text search "unconditional cash transfer"	02/11/2017
**DFiD**	free text search "unconditional cash transfer"	02/11/2017
**SSRN**	free text search "unconditional cash transfer"	31/10/2017
**Socialprotection.org**	applied filters 'unconditional cash transfer' and 'publication'	01/11/2017
**Institute for Poverty Action**	cash transfer page - studies tab	06/11/2017
**ILO**	free text search "unconditional cash transfer" with 'publication' filter applied	02/11/2017

Specialist database searches – update

**Database**	**Search parameters**	**Date**
**EconPapers**	"unconditional cash transfer" working papers, last 3 years	04/07/2019
**EconPapers**	"tribal casino", working papers, last 3 years	30/07/2019
**EconPapers**	"alaska permanent fund", working papers, last 3 years	30/07/2019
**SSRN**	"unconditional cash transfer", title, keywords, abstract, text, last 2 years	24/07/2019
**SSRN**	"basic income", title, keywords, abstract, last 2 years	24/07/2019
**SSRN**	tribal casino	30/07/2019
**SSRN**	alaska dividend	30/07/2019
**Socialprotection.org**	"unconditional cash transfer" publications	04/07/2019
**Socialprotection.org**	"basic income" publications	24/07/2019

Characteristics of studies

Supplementary table 3 provides an overview of the number and type of studies for each intervention. Detailed study and intervention characteristics are provided in Supplementary tables 4-7.

Study design/intervention	NIT	APF	NA casinos	ITSP	OBIP	Total
RCT	7	0	0	0	0	7
Difference studies	2	6	1	1	0	10
Interrupted time series	1	1	1	0	0	3
Synthetic control	0	1	0	0	0	1
Controlled before and after	0	0	1	0	0	1
Qualitative	2	0	3	0	2	7
Total	12	8	6	1	2	29*

*Supplementary table 3 Interventions and study designs. *Two mixed method studies are double counted.*

Historical Negative Income Tax Studies (1968-1980)

When the NIT experiments began, there was widespread political and academic support for the concept of negative income tax, both due to the perceived work and marriage disincentives inherent in the existing welfare system,[3] and in the context of President Nixon's attempts to eradicate poverty.[4] Nonetheless, they faced sustained opposition due to the putative work disincentive effects, which at times hindered the implementation and evaluation of the interventions.[5,6] While they occurred in the USA and Canada in the 1970s, aspects of their contexts were rather similar to current conditions, in that employment insecurity, lone parenthood, and married women's employment were common among the samples.[7-10]

NIT provided scattered samples of low-income families with an unconditional, subsistence-level income that was withdrawn at varying rates above varying income thresholds. All the studies were originally evaluated using RCTs, with samples of households dispersed across large areas. The availability of other welfare benefits, and thus both the control condition and the attractiveness of NIT to experimental respondents, varied widely within, between, and sometimes during the studies. SIME/DIME had one 5 year arm, and a very small sample initially enrolled for 20 years, but otherwise the experiments ran for 3 years. Mincome, in Canada, had dispersed intervention and control groups, and also one saturated site in the town of Dauphin, where anyone whose income fell below the threshold during the study was eligible. Early analyses conducted by the study researchers included only the two dispersed samples.[11] Recent analyses use quasi-experimental and qualitative methods to analyse the Dauphin sample, with the dispersed groups of matched respondents from the surrounding communities acting as controls, thus allowing investigation of community-level effects.[11-14]

The studies have been criticised for various methodological shortcomings,[14] but many of these were addressed by the study authors. The impact of attrition on estimates of annual hours worked was tested using administrative data, and found not to bias the estimates.[16,17] Underreporting of earnings was checked in the same way, and in Gary and SIME/DIME showed that annual hours worked did not decrease by as much as the study data suggested.[7,18] The method of allocation was exceedingly complex and increased the probability that higher income respondents would be assigned to more generous intervention arms. Attempts to account for this suggest that the estimates were not affected, but it is not clear whether they account fully for the imbalances built into the randomisation procedure. [16,17] However, multiple variations of the intervention and demographic subgroups led to small sample sizes and weakened the findings.

Contemporary studies

Iranian targeted subsidy plan (2010-)

The Iranian government has paid all individuals a fixed monthly sum since 2010, to compensate for the abolition of fuel subsidies. This was initially higher than the monthly expenditure of 2.8 million Iranians, but inflation reduced the value by two thirds within 7 years.[19,20] It was intended to be permanent, but very few believed that this would be the case,[21] which may have influenced labour market responses. One study used difference in difference (DiD) and fixed effects models to estimate the impact on labour market outcomes, but

these were very short-term. The revenues raised by reforming fuel subsidies were insufficient to cover the costs of the transfer, which ran at a deficit of 1.1% of GDP and was funded by printing money.[19] It is widely believed to have work disincentive effects,[22] and President Rouhani's 2018-19 budget proposed to target payments at people on very low incomes.

Alaska Permanent Fund (1982-)

Distribution of dividends from Alaska's oil resources via the APF is extremely popular with Alaskans, who view it as a right based in their common ownership of oil resources.[23] The APF was implemented in conjunction with the abolition of state level income taxes, meaning that transfers are not recovered from wealthier people via the tax system, and the overall effect of the programme is regressive.[24] If receipt of APF causes anyone to lose eligibility for federal welfare benefits, the state provides an extra payment to cover the loss.[25] The impacts of the APF were evaluated using six DiD studies, one interrupted time series, and one synthetic control. The identification strategy used in each of the DiD studies varies, and the synthetic control study has been criticised for not fully accounting for the idiosyncratic nature of the Alaskan labour market.[26]

Tribal dividends (1990s-)

Approximately 120 Native American nations pay individual members dividends on the proceeds from casinos run on tribal land.[27] Typically, young adults receive most of their childhood payments as a lump sum on reaching the age of 18 or 21.The density of Native Americans living in a given area varies, so the potential for community-level effects is likely to be variable. Casinos are often accompanied by substantial investments in infrastructure and services, as well as increased employment opportunities and college scholarships. The Great Smokey Mountains Study (GSMS) of the Eastern Cherokee nation began before dividends were introduced, and evaluates their effects using a range of QE designs including DiD, triple difference, and fixed effects. GSMS pre-dates infrastructure investment on the reservation, so the effects are not confounded. However, the study is limited by small sample sizes; the full sample of Native American children is 350. A further study of the Eastern Cherokee used both DiD and ethnographic methods. Two studies of tribal dividends used qualitative methods to evaluate the effects of casino dividends; in the Meskwaki nation, monthly payments are high, and there has been major investment in education, health and housing. In California, not all tribes have casinos, and those that do distribute dividends to all tribes in order to reduce intra-tribe income inequality. One controlled before-and-after study compared outcomes among Native American Nations without casinos, with casinos but no dividends, and with casino dividends.

Ontario Basic Income Pilot (2018-19)

OBIP was very similar in design to the earlier Canadian Mincome study; an NIT which was paid at a household level to working age individuals or couples whose income fell below a given threshold (Can$34,000 for a single person, Can$48,000 for a couple), and which was withdrawn at a rate of 50% on any income above Can$16.989 for a single person and Can$24,027 for a couple. Payments were to have been made for a period of 3 years. Additional payments were available to people with disabilities, up to a maximum of Can$500 per month. It was not conditional on working or seeking employment and was available to people in education. The researchers were unable to use administrative systems to identify low-income respondents, forcing them to attempt recruitment via a mail-out to the general population. This was so unsuccessful they resorted to recruiting respondents through community organisations, introducing a high level of selection bias.[28] The pilot was ended early when a Conservative administration won the provincial election in 2018. Two studies have collected qualitative data from participants, but the sampling methods are likely to further increase selection bias.

Basic income criteria

Intervention	unconditional	universal	permanent	subsistence	fixed
North American NIT	y	n	n	y	n
Mincome saturated site	y	QU	n	y	n
APF	y	y	y	n	y
Iran targeted subsidy plan	y	y	y	y	y
Native American casinos	y	QU	y	varied	y
OBIP	y	n	n	y	n

Supplementary table 2 Basic income criteria met by included interventions.
QU= quasi-universal

no
yes
partially

The above table shows which of the basic income criteria each of the interventions met. We defined interventions in which a large proportion of the population could receive payments as quasi-universal, because spillover or higher-level effects are likely to occur in situations where there is a high density of recipients and/or all members of the community know that they could receive payments if necessary. For instance, although Native American casino dividends are not truly universal, tribes are discrete political units, and all members of the tribe are eligible for payments. In Mincome's Dauphin site, only people with income below the threshold qualified, but anyone whose income fell below the threshold during the study could receive payments.

References

1. Gibson M, Hearty, W., Craig, P., Watson N. Review protocol. The impact of Universal Basic Income: A scoping review of evaluations and outcomes. Glasgow: What Works Scotland, 2017.
2. Widerquist K. A Failure to Communicate: The Labor Market Findings of the Negative Income Tax Experiments and Their Effects on Policy and Public Opinion: US Basic Income Guarantee, 2002.
3. Greenberg DH, Donna L, Marvin M. Social experimentation and public policymaking. Washington, D.C.: The Urban Insitute; 2003.
4. Munnell AH. Lessons from the Income Maintenance Experiments: An Overview. In: Munnell AH, ed. Lessons from the income maintenance experiments: Proceedings of a conference held in September 1986. Boston: Federal Reserve Bank of Boston and the Brookings Institution; 1986.
5. Kershaw D, Fair J. New-Jersey Income-Maintenance Experiment Vol. 1. Operations, Surveys, And Administration. New York: Academic Press Inc.; 1976.
6. Widerquist K. A failure to communicate: What (if anything) can we learn from the negative income tax experiments? *Journal of Socio-Economics* 2005; **34**(1): 49-81.
7. Burtless G. The Work Responses To A Guaranteed Income: A Survey of Experiment Evidence. In: Munnell AH, ed. Lessons from the income maintenance experiments: Proceedings of a conference held in September 1986. Boston: Federal Reserve Bank of Boston and the Brookings Institution; 1986.
8. Choudhry SA, Hum DPJ. Graduated work incentives and how they affect marital stability - the Canadian evidence. *Appl Econ Lett* 1995; **2**(10): 367-71.
9. Christophersen G. Final report of the Seattle-Denver income maintenance experiment Volume 1: Design and Results. Princeton: Mathematica Policy Research, Inc; 1983.
10. Kelly TF, Singer L. Gary income maintenance experiment - plans and progress. *Econometrica* 1971; **39**(4): 358-&.
11. Calnitsky D, Latner JP. Basic Income in a Small Town: Understanding the Elusive Effects on Work. *Social Problems* 2017; **64**(3): 373-97.
12. Calnitsky D. "More Normal than Welfare": The Mincome Experiment, Stigma, and Community Experience. *Canadian Review of Sociology* 2016; **53**(1): 26-71.
13. Forget EL. The Town with No Poverty: The Health Effects of a Canadian Guaranteed Annual Income Field Experiment. *Canadian Public Policy-Analyse De Politiques* 2011; **37**(3): 283-305.
14. Forget EL. New questions, new data, old interventions: The health effects of a guaranteed annual income. *Preventive Medicine* 2013; **57**(6): 925-8.
15. Y Combinator Research. Basic Income Project Proposal. Oakland, CA, 2017.
16. Hum D, Simpson W. Economic response to a guaranteed annual income: experience from Canada and the United States. *Journal of Labor Economics* 1993; **11**(1, Part 2): S263-S96.
17. Robins PK, West R. Part III Chapter III. General Evaluation Issues. In: SRI International, ed. Final report of the Seattle-Denver income maintenance experiment Volume 1: Design and Results. Washington, D.C: U.S. Dept. of Health and Human Services; 1983.
18. Greenberg D, Halsey H. Systematic misreporting and effects of income-maintenance experiments on work effort - evidence from the Seattle-Denver experiment. *Journal of Labor Economics* 1983; **1**(4): 380-407.
19. Salehi-Isfahani D. Iran's subsidy reform: From promise to disappointment: Economic Research Forum (ERF), 2014.
20. Salehi-Isfahani D. Tyranny of numbers. Rouhani's new budget aims to eliminate cash transfers 2017. https://djavadsalehi.com/2017/12/31/rouhanis-new-budget-aims-to-eliminate-cash-transfers/#more-4119 (accessed 5th February 2018).
21. Tabatabai H. Iran: A Bumpy Road toward Basic Income. Basic Income Guarantee and Politics: International Experiences and Perspectives on the Viability of Income Guarantee; 2012: 285-300.
22. Salehi-Isfahani D, Mostafavi-Dehzooei MH. Cash transfers and labor supply: Evidence from a large-scale program in Iran. *Journal of Development Economics* 2018.
23. Widerquist K, Sheahen A. The United States: The Basic Income Guarantee — Past Experience, Current Proposals. In: Murray MC, Pateman C, eds. Basic Income Worldwide: Horizons of Reform. London: Palgrave Macmillan UK; 2012: 11-32.
24. Zelleke A. Basic Income and the Alaska Model: Limits of the Resource Dividend Model for the Implementation of an Unconditional Basic Income. Alaska's Permanent Fund Dividend: Examining Its Suitability as a Model; 2012: 141-55.
25. Bibler A, Guettabi M, Reimer MN. Short-term Labor Responses to Unconditional Cash Transfers. Anchorage: Institute of Social and Economic Research., 2019.
26. Feinberg RM, Kuehn D. Guaranteed Nonlabor Income and Labor Supply: The Effect of the Alaska Permanent Fund Dividend. *The BE Journal of Economic Analysis & Policy* 2018; **18**(3).

27. Akee R, Simeonova E, Costello EJ, Copeland W. How does household income affect child personality traits and behaviors? Cambridge, MA: National Bureau of Economic Research, 2015.
28. Mason G. It was time to walk away from Ontario's flawed basic income project. [online] 2019. Available at: https://theconversation.com/it-was-time-to-walk-away-from-ontarios-flawed-basic-income-project-101217 (accessed 1st November 2019).

Intervention and study characteristics tables

Study/ intervention	Location/s	Dates	Duration	Population	Level of implementation	Household/ individual	Income threshold	Guarantee level and withdrawal rate	Co-interventions
**Gary Income Maintenance Experiment**	Gary, Indiana	1971-74	3 years	Black families with at least one child under the age of 18. 60% welfare dependent female headed families	Individual, scattered sample	Household	Below 240% of poverty line	Provided subsistence level income for those with no other income source.<br>Four combinations of guarantee and tax rate<br>(1) 75% of poverty line guarantee, 40% tax rate;<br>(2) 75% and 60%;<br>(3) 100% and 40%;<br>(4) 100% and 60%	Other treatments were initially provided (access to social services, day care) but terminated due to undersubscription
**Manitoba Basic Annual Income Experiment**	Winnipeg, Manitoba scattered sample site, Dauphin, Manitoba saturation site	1975-79	3 years	Low-income households with able-bodied heads under 58 years of age. Included single people with no children (21% of all men in the sample were single)	Individual; two scattered samples and one saturation site	Household	Below 150% of poverty line	Provided subsistence level income for those with no other income source.<br>8 combinations of maximum benefit level and tax rate (CAD $)<br>(1) 3800, 35% (2) 4,800, 35%<br>(2) 3,800, 50% (2) 4,800, 50%<br>(2) 5,800, 50% (2) 3,800, 75%<br>(2) 4,800, 75% (2) 5,800, 75%<br><br>The Dauphin saturation site only tested one combination. Anyone whose income fell below the threshold during the study was eligible for Mincome and joined the study sample.	None
**New Jersey Graduated Work Incentive Experiment**	Trenton, Jersey City, and Paterson, New Jersey, and Scranton, Pennsylvania	1968-72	3 years	Black, white, and Latino, two-parent families in urban areas with a male nondisabled head aged 18–58, and at least one dependent; unclear whether this had to be a child	Individual, scattered sample	Household	Below 150% of poverty line	Provided subsistence level income for those with no other income source.<br>Eight combinations of guarantees and tax rates<br>(1) 50% of poverty line guarantee, 30% tax rate<br>(2) 50% guarantee, 50% tax rate;<br>(3) 75% guarantee, 30% tax rate;<br>(4) 75% guarantee, 50% tax rate;<br>(5) 75% guarantee, 70% tax rate;<br>(6) 100% guarantee, 50% tax rate;<br>(7) 100% guarantee, 70% tax rate;<br>(8) 125% guarantee, 50% tax rate	None
**Rural Income Maintenance Experiment**	Duplin County, North Carolina, and Pocahontas and Calhoun Counties, Iowa	1969-72	3 years	Rural, low-income families, in which the male head was 18-58 and not disabled. Female headed and aged or disabled headed households were also sampled but excluded from analyses because numbers were too low	Individual, scattered sample	Household	Below 150% of poverty line	Provided subsistence level income for those with no other income source.<br>Five combinations of guarantees and tax rates<br>(1) 50% of poverty line guarantee, 50% tax rate;<br>(2) 75% guarantee, 30% tax rate;<br>(3) 75% guarantee, 50% tax rate;<br>(4) 75% guarantee, 70% tax rate;<br>(5) 100% guarantee, 50% tax rate	None

Study/ intervention	Location/s	Dates	Duration	Population	Level of implementation	Household/ individual	Income threshold	Guarantee level and withdrawal rate	Co-interventions
Seattle/Denver Income Maintenance Experiment	Seattle, Washington, and Denver, Colorado	1970-80	3, 5 and 20 years	Couples (either married or cohabiting, and with or without children) or single heads of households with at least one dependent child younger than 18 and including a nondisabled husband or a single, nondisabled, female head of household, 18-58 years old. Black and White in Seattle, Black, White and Latino in Denver.<br>40% were single parents	Individual, scattered sample	Household	$9000 p.a. for single worker, or $11000 for two worker households	Provided subsistence level income for those with no other income source. Tapered withdrawal rate which changed for each £1000 of earned income. The change in withdrawal rate is listed last in the following:<br>11 combinations of guarantee, tax rate, and change in tax rate for $1000 income increase:<br>(1) 95%,50%,0%; (2) 95%, 70%, 0%; (3) 95%, 70%, -2.5%; (4) 95%, 80%, -2.5%;<br>(5) 120%,50%,0%; (6) 120%, 70%, 0%; (7) 120%, 70%, -2.5%; (8) 120%, 80%, -2.5%; (9) 140%,50%,0%; (10) 140%, 70%, 0%; (11) 140%, 80%, -2.5%	3 co-interventions were included in the study:<br>Free vocational counselling<br>Free counselling plus a 50% tuition subsidy for education or career-related training<br>Free counselling plus a 100% tuition subsidy

Supplementary table 4 Negative Income Tax intervention characteristics

Study	Study design	Data source	Sample size at baseline	Study dates	Comparison group/identification strategy	Analysis method	Methodological issues	Implementation	Context
**Gary Income Maintenance Experiment**	RCT	Study data	Total n=1799	1971-75	Usual care - no welfare for 2 parent families, available to single parent families	The analyses conducted by NIT research staff used a range of methods, but most appear to be regression or ANOVA based	Conlisk-Watts method of allocation not used. However, there were a number of other issues with representativeness of the sample	The involvement of state level authorities in delivering the programme facilitated implementation	NIT was much more generous than existing welfare payments.<br><br>Specifically focused on black female-headed families in urban "ghetto" settings - 60% of sample. Labour market dominated by steelworks, opportunities for part-time or hours reduction limited
**Manitoba Basic Annual Income Experiment**	RCT	Study data	Sources conflict; total n appears to be around 1700	1975-79	Usual care - Income Assistance, eligibility conditions unclear. Dauphin saturation site was not included in early analyses	ANOVA methods	Conlisk-Watts method of allocation<br><br>Attrition 36%, but this does not appear to have biased the estimates	See Calnitsky 2016	Urban and rural areas of the Canadian prairies
**Manitoba Basic Annual Income Experiment (Calnitsky 2016)**	Qualitative	Study survey data; open-ended question responses	321 Mincome recipients, 98 non-recipients and 40 Manitoba welfare recipients	1976	40 surveys completed by Manitoba welfare recipients and 98 surveys completed by low income non-recipient "controls" from various small Manitoba towns. Income Assistance available to intervention and control groups	Line-by-line and axial coding	Based on an analysis of responses to open-ended survey questions. Only a small proportion of respondents completed these questions. Potential selection bias is not addressed. Some analyses are based on very small samples	A number of respondents noted that Mincome staff were more respectful than mainstream welfare services	Attitudes towards Mincome were very positive. Mincome was seen as morally superior to normal welfare. Application process was also much less onerous and stigmatising
**Manitoba Basic Annual Income Experiment (Calnitsky and Latner 2017)**	QE; DiD, qualitative	Study data	Saturation sample n=47 (Dauphin), scattered sample n=45 (Manitoba treatment), control n=100 (Manitoba control)	1973-78	Dispersed treatment and control study samples in Manitoba. Income Assistance available to intervention and control groups	Regression modelling	Only respondents with no missing data in two separate study datasets are included in the analysis. This reduces the sample from 1050 to 292. Potential selection bias is not addressed. Some analyses are based on very small samples	See Calnitsky 2016	See Calnitsky 2016
**Manitoba Basic Annual Income Experiment (Calnitsky 2018)**	DiD	Survey of business owners	Intervention n = 292, control n = 1155	1974-75	7 rural control towns; unclear how these were selected	Regression modelling	Low response rate and short follow-up (9 months). Control group selection unclear. Potential response bias for social or political reasons	See Calnitsky 2016	Dauphin labour market was seasonal and precarious
**Manitoba Basic Annual Income Experiment (Forget 2011, 2013, 2013a)**	QE; Interrupted time series	Routine data	Ns not reported. Intervention group = all continuous residents of Dauphin from 1974-1979. Control = 3 Manitoba residents per intervention group member	1971-85	Multiple controls selected from surrounding rural areas using propensity score matching. Income Assistance available to intervention and control groups	Negative binomial distribution model	The analyses include all residents of Dauphin, whether they received/ were eligible for Mincome. It is not possible to tell whether effects were concentrated in recipients (30%) or spread across the whole sample	See Calnitsky 2016	All Dauphin residents whose income fell below the threshold were eligible for Mincome. 30% of Dauphin's population received Mincome at some point during the study, but payments were typically small

Study	Study design	Data source	Sample size at baseline	Study dates	Comparison group/identification strategy	Analysis method	Methodological issues	Implementation	Context
**New Jersey Graduated Work Incentive Experiment**	RCT	Study data	Total n=1216	1968-72	Usual care - welfare available to 2 parent families (initially more generous than most NIT plans, later reduced dramatically)	The analyses conducted by NIT research staff used a range of methods, but most appear to be regression or ANOVA based	Conlisk-Watts method of allocation<br><br>Differential attrition - higher earners had less incentive to stay in the study, as they no longer qualified for benefits; there is some evidence that non-attriters had 'higher preference for leisure', but this does not appear to have biased the estimates	State govts were not involved in delivering the NIT, which caused many problems for the families and the staff.<br><br>Early contact with the media generated negative publicity and led to some respondents being harassed	Declining urban industrial areas.<br><br>Welfare for two parent families implemented soon after intervention started, both groups eligible. Most experimental families eligible for lower level guarantees claimed welfare. Welfare also passported to Medicaid and food stamps. Value was substantially reduced in 1971.<br><br>The experiment generated a great deal of controversy at the time, leading to calls for its cessation due to suspicions of fraud
**Rural Income Maintenance Experiment**	RCT	Study data	Total n=809; 587 male headed families retained in primary analyses	1969-73	Usual care - no welfare for 2 parent families	The analyses conducted by NIT research staff used a range of methods, but most appear to be ANOVA regression based	Conlisk-Watts method of allocation<br><br>Small sample size, further stratified to include single female headed households, households with an aged head, farm and non-farm residents, as well as across 2 very distinct geographical areas. Few estimates significant and generalisability very limited	State govts were not involved in delivering the NIT, which caused many problems for the families and the staff.<br><br>Policy makers were keen to include many different subgroups in the study. However, the resulting samples were too small for meaningful analysis	Conducted in rural areas and sample included farm and non-farm recipients. North Carolina was an area of high poverty with a predominantly black population, while Iowa was relatively affluent and predominantly white
**Seattle/Denver Income Maintenance Experiment**	RCT	Study data	Total n=4800 169 Denver families enrolled in the 20 yr. sample in 1974; terminated in 1980	1970-80	Usual care - no welfare for 2 parent families, available to single parent families	The analyses conducted by NIT research staff used a range of methods, but most appear to be regression or ANOVA based	Conlisk-Watts method of allocation<br><br>Large number of treatment groups greatly weakened the precision of the estimates. Allocation method was particularly complex due to different duration of treatment groups and inclusion of training/counselling treatment groups	The involvement of state level authorities in delivering the programme assisted with implementation. NIT recipients were eligible for passported benefits	The aerospace industry dominated the Seattle economy and it was experiencing a severe recession at the time of the study. The Denver study site was chosen to provide a more representative and stable labour market
**Seattle/Denver Income Maintenance Experiment (Price 2016)**	RCT	Study and routine data	Total n=2280	1978-2013	Usual care - no welfare for 2 parent families, available to single parent families	Least squares regression	In the absence of interim data, it is difficult to attribute any differences to the intervention after such a long period (40 years)	See Seattle/Denver Income Maintenance Experiment	Forty years after the initial intervention
**Seattle/Denver Income Maintenance Experiment (Stephens 2007)**	RCT	Study data	Total n=1923	1970-80	Usual care - no welfare for 2 parent families, available to single parent families	Regression modelling	See Seattle/Denver Income Maintenance Experiment	See Seattle/Denver Income Maintenance Experiment	SIME/DIME had NIT and job training interventions, both of which could run for 3 or 5 years. Previous analyses accounted for the variation in NIT duration, but not the variation in job training

Study	Study design	Data source	Sample size at baseline	Study dates	Comparison group/identification strategy	Analysis method	Methodological issues	Implementation	Context
DiD = difference-in-difference QE = quasi-experiment RCT = randomised controlled trial									
Negative Income Tax study characteristics									

Intervention	Location	Intervention dates	Duration	Type of programme	Population	Implementation level	Household/ individual	Income threshold	Value of payment	Co-interventions
**Alaska Permanent Fund**	Alaska	1982	Ongoing	resource dividend	Whole population of Alaska	National	Individual	n/a	Variable; individuals' payments have ranged from $1000 to $2072 between 1996 and 2015. Value for a family of 4 can be equivalent to federal benefit entitlements	Alaska abolished state level taxes when APF was implemented.<br><br>If APF disqualifies a family from receiving other benefits, APF makes good the shortfall
**Californian Native American nations tribal dividend**	California, USA	Various	Ongoing	tribal dividend	American Indian gaming communities	Population can be concentrated on reservation land, but some tribal members live outside reservation	Individual	n/a	Varied across Native American nations. No detail reported	Investment in health and social services on reservations. Most young adults receive a large lump sum at 18
**Eastern Cherokee Nation tribal dividend**	11 counties in North Carolina, USA	1996-	Ongoing	tribal dividend	All members of Eastern Cherokee tribe - historically high poverty and unemployment on average	Population can be concentrated on reservation land, but some tribal members live outside reservation	Individual	n/a	Initially approximately $4,000 per person annually; $9,000 a year by 2006. Represented 20-40% of households' income. Paid biannually. Children's cash transfers are banked for them until age 18	Investment in health and social services on reservation was subsequent to data collection. All young adults receive large lump sum at 18 (approx. $30,000 in 2011) or 21 if they do not have a high school degree
**Iranian subsidy reform plan**	Iran	2010-	Ongoing at time of writing	fuel subsidy reform	Whole population of Iran	National	Household	n/a	Approximately $90 pcm. Initially represented 29% of median household income	None reported
**Meskwaki Nation tribal dividend**	Iowa, USA	Mid 1980s-	Ongoing	tribal dividend	All members of Meskwaki tribe - historically high poverty and unemployment on average	Population can be concentrated on reservation land, but some tribal members live outside reservation	Individual	n/a	$1800 pcm. 75% of children's cash transfers are banked for them until age 18 if they complete high school leaving exams. 25% is paid to parents	Investment in health, housing, and social services on reservation. All young adults receive large lump sum at 18 of up to $200,000
**Native American Nations tribal dividend**	USA	Various	Ongoing	tribal dividend	All members of Native American gaming tribes	Population can be concentrated on reservation land, but some tribal members live outside reservation	Individual	n/a	Varied across Native American nations. No detail reported	Investment in health and social services on reservations. Most young adults receive large lump sum at 18
**OBIP**	Ontario, Canada	2018	12 months	NIT	Working age people	Individual; two scattered samples and one saturation site	Individual	Can$34,000 for a single person, Can$48,000 for a couple	75% of the Low Income Measure (Can$16.989 for a single person and Can$24,027 for a couple). 50% withdrawal rate for any income above this	No formal interventions, but respondents started a community organisation which many respondents became involved in
Contemporary intervention characteristics										

Intervention	Study	Study design	Data source	Sample size at baseline	Study dates	Comparison group/identification strategy	Analysis method	Methodological issues	Implementation	Context
**APF**	Chung 2017	QE: DiD	Routine data	7·7 million live births (52,346 in Alaska)	1978-1984	44 US states	Regression model	None known	Not reported	See Kozminsky 2017
**APF**	Evans 2011	QE: DiD	Routine data	Not reported; all US deaths during study period	2000-2006	Other US states	Regression model	None known	Not reported	See Kozminsky 2017
**APF**	Jones 2018	QE; Synthetic control	Routine data	Employment data: 48,686,169 observations for the study period	1977-2015	Synthetic control - weighted average of control states to best match Alaska for the outcome of interest and other observable characteristics before the dividend payments began	Synthetic control	Identification strategy does not account for the more buoyant Alaskan labour market post-1982 when the rest of the county was in recession[25]	Not reported	See Kozminsky 2017
**APF**	Kozminsky 2017	QE; Interupted time series	Routine data	Annual observations of income inequality for all US states during the study period	1963-2012	Time series data 1963-2012	Autoregressive distributed lag (ARDL) and the Johansen co-integration approach to ITS	None known	Not reported	Interaction with the tax system means APF is regressive, as payments are not recovered from higher earners.<br><br>If APF disqualifies a family from receiving other benefits, APF makes good the shortfall
**APF**	Bibler 2019	QE: DiD	Current Population Survey	Observations: Men high/low PFD years: 28027/ 32808 Women high/low PFD years:30742/ 37580	1994-2012	Annual variation in the size of PFD payments to identify effects, dichotomized into low and high PFD years	Unclear	No information is provided on the survey dataset used, nor on the sample size, therefore representativeness is unclear, but the sample only includes people aged 20-55 who are heads of households or spouses of same.	APF is paid to Green Card holders and refugees	"This cash transfer is particularly important in rural areas where economies lack economic bases and are still a mixture of subsistence and a small formal economy."
**APF**	Feinberg 2018	QE: DiD	American Community Survey (ACS). State unemployment data and other sources	For models including all states: Men 12,950,193 Single women 7,019,223 Married women 7,000,428	2005-15	Comparison states are Montana and Hawaii. Also run models using all US states.<br><br>Also use ineligible Alaskans as a comparison group	Unclear	They note that the correlation of family size with APF size is problematic, as the number of children in the household also affects mothers' propensity to work<br><br>Alaskan labour market differs a great deal from other US states	APF is not counted as income when assessing eligibility for Medicaid, food stamps, or any other federal assistance programmes	The value of the APF is not dictated by the oil price as revenues are invested

Intervention	Study	Study design	Data source	Sample size at baseline	Study dates	Comparison group/identification strategy	Analysis method	Methodological issues	Implementation	Context
**APF**	Watson 2019	QE: DiD	Alaska Longitudinal Child Abuse and Neglect Linkage Project	n=880	2009-14	Variations in amount of dividend received due to fluctuating annual value and child birth date	Unclear	Authors note there are issues with systematic non-response bias and also measurement error	The study focuses on payments in respect of children; authors note evidence such payments tend to be used for children's needs	Studied population is younger and has higher baseline obesity than previous studies
**APF**	Watson 2019a	QE: DiD	All police incident reports for Anchorage Alaska.		2000-16	Compare crime rates on days/weeks following payment receipt to similar periods with no cash transfer. Also use annual variation in value of transfer	Unclear	None known	Payments are made as annual lump sums, which may lead to a larger impact than staggered payments would have	No additional information
**Iranian subsidy reform plan**	Salehi-Isfahani 2018	QE; DiD, fixed effects	Routine data	Total n=37,751	2010-11	30% of the population received payments 3 months later than the remainder. This group was used as a control in the DiD analyses. Households where the payment represented a smaller proportion of household income were used in the fixed effects analyses	Regression modelling	High attrition from the survey (45%); weighting used to correct for this	It was initially intended that the cash transfer would be targeted at poorer people, but this was scrapped due to protests and because targeting proved too complex to administer	There was opposition to the policy due to its anticipated work disincentive effect.<br><br>The majority of people did not believe that the programme would be permanent.<br><br>Very high inflation rapidly eroded the value of the payments.<br><br>Only 18% of Iranian women are in employment
**OBIP**	BICN 2019	Survey with open ended questions	Bespoke survey conducted by advocacy group	424	2018	n/a	Not reported	Little methods information is provided. There were very serious selection bias issues with the planned study. The survey sample is self-selected from an already biased larger sample.	There were many problems with study recruitment. The study was terminated after only one year due to a change of provincial government	OBIP payments were considerably higher than existing welfare entitlements
**OBIP**	Hamilton 2019	Qualitative	One to one interviews	5	2018	n/a	Phenomenological data analysis	There were very serious selection bias issues with the planned study. The qualitative sample is very small and drawn from people participating in a vocal community group. Selection bias likely to be very high	There were many problems with study recruitment. The study was terminated after only one year due to a change of provincial government	OBIP payments were considerably higher than existing welfare entitlements
**Tribal dividends**	Bruckner 2010	QE; Interrupted time series. Ethnography	Routine data	Total population of study areas = 57000	1990-2006	Monthly data series from 1990-2006	Poisson regression	Due to the low death rate in the sample population, the analysis sample was small (n=75).<br><br>Results from an ethnographic study reported but no methods information is provided	Results suggest that the timing and value of payments influences effects on mortality	Young people receive a large lump sum on reaching 18. There is evidence this is often spent on vehicles and substances

Intervention	Study	Study design	Data source	Sample size at baseline	Study dates	Comparison group/identification strategy	Analysis method	Methodological issues	Implementation	Context
**Tribal dividends**	Conner 2013	Controlled before and after	Routine data	330 Native American nations in 32 US states	1990-2000	Nations without gaming, Class II (low stake) gaming nations, class III (high stake) gaming nations without per capita payments	Robust regression	The sample of Native American nations is small, particularly for subgroup analysis. There are no data on intervention characteristics within each nation. There are quite a lot of missing data	Not reported	The introduction of casinos has been accompanied by large scale investment in health care, housing, and other services. However these are controlled for by comparing Class III gaming nations with and without per capita payments
**Tribal dividends**	Foley 2005	Qualitative	Interviews	60 tribal members active in tribal affairs and 20 white business people, educators, and casino employees	1995-2004	None	Not reported	Methods are not clearly described. Method of recruiting key actors unclear.<br><br>Qualitative findings in small communities may be influenced by salience bias	There are issues around who is defined as a 'pure blood' tribal member for establishing eligibility, which can lead to conflict	Meskwaki payments are particularly large, and young people receive a very large lump sum on reaching 18. There is also very substantial investment in housing, infrastructure and services, and college education is paid for
**Tribal dividends**	Great Smoky Mountains Study	QE; DiD, triple difference, controlled before and after	Study data	1,420 children aged 9, 11, and 13 years at baseline (350 Native American, 1070 non-Native American)	1993-2003	Non-Native American children (in households not receiving dividends), older cohorts of Native American youth. 10 waves of data collected during study; ages and length of exposure vary across publications	Linear and panel fixed-effects regression, marginal model approach (generalized estimating equations applied to the longitudinal data) (5 publications using different methods)	The samples were small	Not reported	There was an upsurge in investment in health and social services following the introduction of tribal casinos. However, the study authors state that this occurred after the study data were collected.<br><br>Young people receive a large lump sum on reaching 18 if they have completed high school
**Tribal dividends**	Kodish 2016	Qualitative	Interviews	12 tribal leaders and 24 tribal members from 23 tribal communities	2014-2015	None	Grounded Theory - line by line coding	Not possible to tell whether respondents are receiving per capita payments, nor how much they are.<br><br>Qualitative findings in small communities may be influenced by salience bias	There appear to be some issues around transparency of dividend management	Sample drawn from 23 of 109 tribes in California.<br><br>Californian tribes operate a system of redistributing dividends to non-gaming tribes
**DiD = difference-in-difference QE = quasi-experiment RCT = randomised controlled trial**										
Contemporary study characteristics										

Intervention	Study	Outcomes reported
**APF**	Chung 2017	Birthweight<br>AGPAR score<br>Length of gestation<br>Number of pre-natal care visits<br>How long receiving pre-natal care
**APF**	Evans 2011	Accidental mortality
**APF**	Hsieh 2003	Consumption - seasonal variations in expenditure **(not reported here)**
**APF**	Jones 2018	Employment rate<br>Labour force participation<br>Part-time employment rate<br>Hours worked last week<br>Male part-time employment rate<br>Female part-time employment rate
**APF**	Kozminsky 2017	Income inequality (Gini Coefficient, Relative Mean Deviation (RMD) and Thiel's Entropy Index)
**APF**	Bibler et al 2019	Number of hours worked in reference week<br>Whether respondent employed in reference week<br>Number of hours worked in reference week (men)<br>Whether respondent employed in reference week (men)<br>Number of hours worked in reference week (women)<br>Whether respondent employed in reference week (women)
**APF**	Watson et al 2019a	Probability of child obesity at age 3<br>Probability of child obesity at age 3, income <$25k<br>Probability of child obesity at age 3, income $25- 75k<br>Probability of child obesity at age 3, income >$75k
**APF**	Watson et al 2019	Noise violations one day after PFD receipt<br>Property crime one day after PFD receipt<br>Substance abuse-related crime one day after PFD receipt<br>Violent crime one day after PFD receipt<br>Medical assistance to other agencies one day after PFD receipt<br>Noise violations four weeks after PFD receipt<br>Property crime in the four weeks after PFD receipt<br>Substance abuse-related crime in the four weeks after PFD receipt<br>Violent crime four weeks after PFD receipt<br>Medical assistance to other agencies four weeks after PFD receipt
**APF**	Feinberg and Kuehn 2018	Annual hours worked (men)<br>Annual hours worked (single women)<br>Annual hours worked (married women)
**APF**	Olson 1990	Utility maximisation **(not reported here)**
**Iranian subsidy reform plan**	Salehi-Isfahani 2018	For men, women, employed and self-employed, by age and sector:<br>Number of hours worked<br>Labour force participation<br>**Not reported here:**<br>Savings<br>Expenditure (per capita, on food, on durables)<br>Income
**Negative Income Tax**	Gary Income Maintenance Experiment	**Impacts discussed in this review:**<br>Adult labour supply: annual hours worked, % difference in annual hours worked<br>Reported for male heads, second earners, female heads, and teens<br>Marital dissolution<br>Reading Test Scores<br>Academic Grade Point Average<br>Days Absent

		Birth weight<br>**Impacts not discussed in this review:**<br>Labour supply- black family heads<br>Labour supply - farm families<br>Labour supply - underreporting<br>Assets/debts<br>Business decisions<br>Housing service use<br>Social service use
**Negative Income Tax**	Manitoba Basic Annual Income Experiment	Adult labour supply: annual hours worked, % difference in annual hours worked<br>Reported for male heads, second earners, female heads, and teens.<br>Marital dissolution
**Negative Income Tax**	Manitoba Basic Annual Income Experiment (Calnitsky 2016)	Reason for joining Mincome<br>Role of perceived stigma in decisions to claim Mincome/standard welfare benefits
**Negative Income Tax**	Manitoba Basic Annual Income Experiment (Calnitsky and Latner 2017)	Labour market participation rate. Reported for multiple subgroups
**Negative Income Tax**	Manitoba Basic Annual Income Experiment (Calnitsky 2018)	Starting wage rate on job (for all job vacancies reported in prior week)<br>Wage rate on job (for persons hired in the past four months)<br>% of businesses which received job applications in last four months<br>% of businesses with no new employees in last four months<br>Hours per week for new employees<br>Hours per week in vacant positions
**Negative Income Tax**	Manitoba Basic Annual Income Experiment (Forget 2011, 2013, 2013a)	Total hospital separations (hospitalisation rate per 1000 people)<br>Hospital separations for "accidents and injuries"<br>Hospital separations for "mental health diagnoses<br>Overall physician claims and physician claims for "mental health diagnoses"<br>Educational participation: % progressing to Grade 11/12 high school<br>Fertility **(not reported here)**<br>Low birthweight<br>Family dissolution
**Negative Income Tax**	New Jersey Graduated Work Incentive Experiment	**Impacts discussed in this review:**<br>Adult labour supply: annual hours worked, % difference in annual hours worked<br>Reported for male heads, second earners, female heads, and teens<br>Marital dissolution<br>Levels of completed education, school enrolment, college attendance<br>Anomy scale, Control of future scale, Community Efficacy Scale, Psychosomatic and Nervous Symptoms Scale, Self-Esteem Scale, Worry Items, Quality of Life, General happiness, Feeling of nothing to do<br>ADULTS: head's and spouse's number of chronic conditions, number of work days lost, number of days spent in a hospital, and number of physician visits.<br>CHILDREN: per capita number of chronic conditions, per capita number of days spent in bed, whether any child has spent at least one night in a hospital in the year previous to the interview, per capita number of visits to a physician<br>**Impacts not discussed in this review:**<br>Labour supply - effects of health on<br>Labour supply - job selection<br>Labour supply - psychological factors<br>Labour supply - risk taking<br>Labour supply - unemployment duration<br>Labour supply - whole family<br>Consumption<br>Family formation<br>Fertility<br>Housing - purchasing<br>Housing - demand<br>Social outcomes<br>Wages
**Negative Income Tax**	Rural Income Maintenance Experiment	**Impacts discussed in this review:**<br>Adult labour supply: annual hours worked, % difference in annual hours worked<br>Reported for male heads, second earners, female heads, and teens

		Marital dissolution<br>Mean Adequacy Ratio of 10 vital nutrients<br>Self-report delinquency scale: how many times in last 2 years committed theft, received stolen property, trespassed, committed assault, extortion, used marijuana or other narcotics. A further scale developed to take account of seriousness of offences<br>Attendance<br>Academic grades and Standardized Achievement Test score<br>Psychological well-being; scales similar to New Jersey<br>**Impacts not discussed in this review:**<br>Labour supply - employees<br>Labour supply - farmers<br>Labour supply - business performance<br>Assets/debts<br>Consumption<br>Employment decision making<br>Mobility<br>Political participation
**Negative Income Tax**	Seattle/Denver Income Maintenance Experiment	**Impacts discussed in this review:**<br>Adult labour supply: annual hours worked, % difference in annual hours worked<br>Reported for male heads, second earners, female heads, and teens<br>Marital dissolution<br>Achievement scores, academic grades, and absence rates<br>Remaining in school<br>(1) number of work days lost due to illnesses, (2) number of hospital stays and (3) number of days hospitalized in the last 2 years, (4) number of work days missed in the last 6 months, (5) presence of a functional limitation on doing household tasks, (6) presence of a chronic condition that limits activities of daily living or (7) market work, (8) the duration of the chronic condition, (9) a mental health index, and (10) self-perception of overall health<br>Psychological distress - "a close variant of the Macmillan Health opinion survey index"<br>**Impacts not discussed in this review:**<br>Labour supply - childcare<br>Assets/debts<br>Consumption<br>Employment decision making<br>Employment transitions<br>Family formation<br>Family life<br>Fertility<br>Housing demand<br>Job satisfaction<br>Marital formation<br>Migration<br>Mobility<br>Occupational choice<br>Time preference
**Negative Income Tax**	Seattle/Denver Income Maintenance Experiment (Price 2016)	Reported for SIME participants and their adult children:<br>Income<br>Labour force participation<br>Disability benefit claims<br>Disability benefit claims % successful<br>Mortality
**OBIP**	BICN 2019	Recipients' accounts of how OBIP affected them, particularly in relation to ability to explore different options, long term planning, health, mobility, and uses of extra payments
**OBIP**	Hamilton and Mulvale 2019	Recipients' accounts of how OBIP affected them, particularly in relation to unconditionality, long term planning, and reduced work disincentives
**Tribal dividend**	Conner 2013	Unemployment (Native Americans Only)<br>Unemployment (all)<br>Labour force participation (Native Americans Only)<br>Labour force participation (all)<br>Per capita income (Native Americans Only)<br>Per capita income (all)

**Tribal dividend**	Bruckner 2010	Accidental mortality
**Tribal dividend**	Foley 2005	Qualitative: community perceptions of impacts of casinos
**Tribal dividend**	Great Smoky Mountains Study	Maternal and paternal labour force participation rate, full-time employment rate<br>Educational attainment (years of completed education at age 21)<br>Finished high school by age 19<br>High school diploma/general equivalency degree by age 19<br>School attendance (days in previous quarter)<br>Young adult obesity<br>Young adult BMI<br>Child health: accidents, allergies, headaches, and eczema<br>Psychiatric disorders amongst children and adolescents - emotional (anxiety or depression), behavioural (conduct or oppositional defiant disorder), and substance abuse disorder·<br>Child and Adolescent Psychiatric Assessment Symptom Score<br>Emotional and behavioural distress<br>Trait conscientiousness<br>Trait agreeableness<br>Trait neuroticism<br>Parental mental health (whether one or both parents sought mental health support)<br>Criminal arrest figures, nature of offence (young adult)<br>Criminal arrest figures (adult)<br>Marital status<br>Parent-child relationship quality<br>Parental supervision<br>Political participation **(not reported here)**
**Tribal dividend**	Kodish 2016	Qualitative: mechanisms linking casinos to health
All outcomes reported by included studies		
